# Supplementary material for: Vps34 PI 3-kinase inactivation enhances insulin sensitivity through reprogramming of mitochondrial metabolism
Source: Nat Commun. 2017 Nov 27;8:1804. doi: 10.1038/s41467-017-01969-4 (PMC5703854; doi:10.1038/s41467-017-01969-4)
Supplement: Supplementary file 2 — Supplementary Information [file 41467_2017_1969_MOESM2_ESM.pdf]

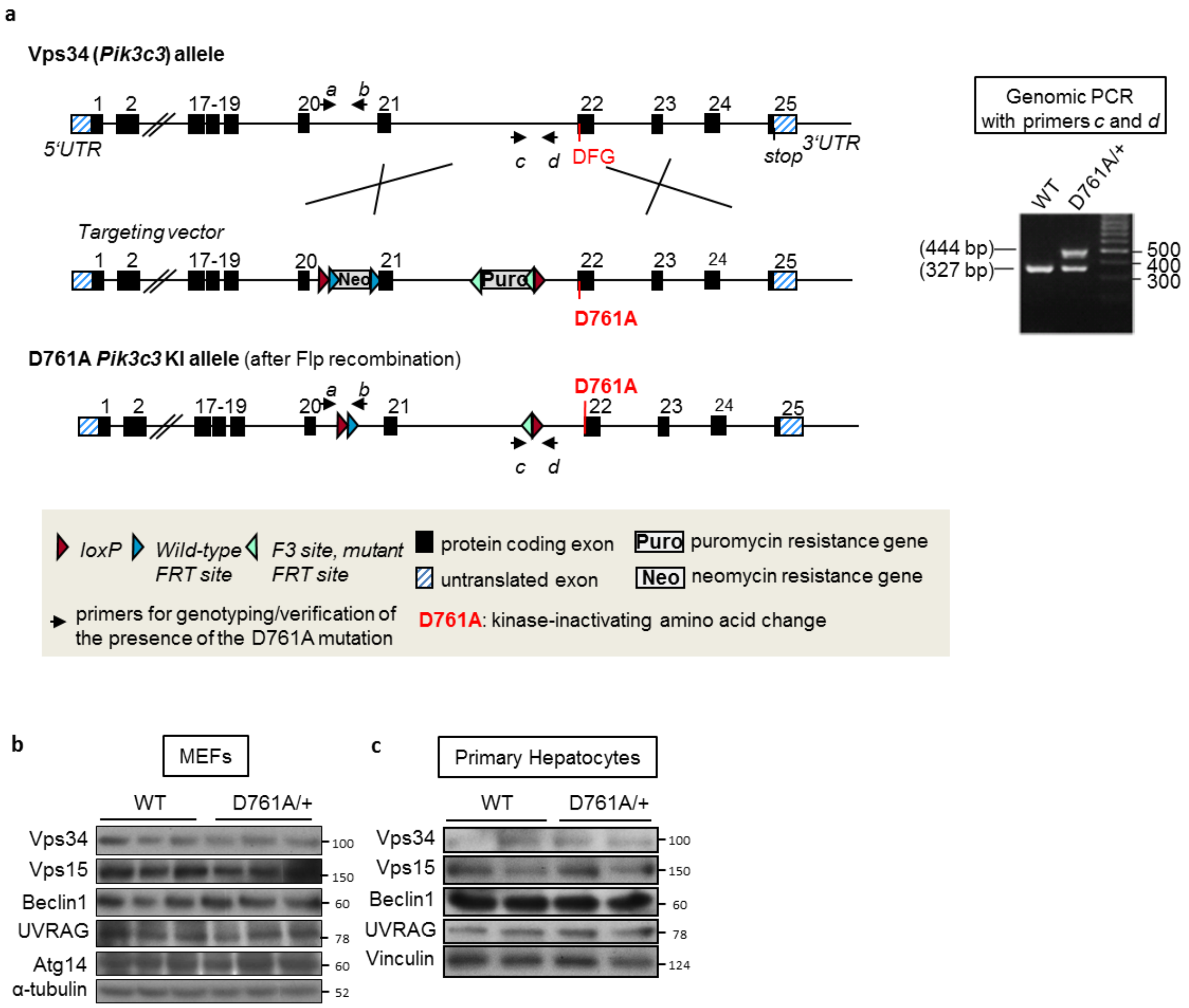

Supplementary Figure 1. Gene targeting and characterization of Vps34$^{D761A/+}$ mice. (a) Gene targeting strategy to generate the constitutive D761A knock-in mutation in the *Pik3c3* gene. The D761A mutation was introduced in the DFG motif in exon 22 of the *Pik3c3* gene. The FRT-flanked cassette encoding the *Pgk Neo* selection marker (Neo) was removed *in vivo* by breeding onto ACTB-Flp mice which express the FLP1 recombinase gene under the control of the *ACTB* (actin) promoter. A representative image of agarose electrophoresis showing PCR products generated using the primer pair 1450_33 and 1450_34 (shown as "a" and "b" in the left panel). (**b, c**) Expression levels of Vps34 binding partners in primary MEFs (**b**) and primary hepatocytes (**c**). Tissue/cell lysates were immunoblotted using the indicated antibodies. 60 µg of protein was loaded per lane. Composite images are derived from experiments whereby equal amounts of the same cell/tissue extract were loaded on separate gels and developed with the same indicated antibodies. This was done in order to quantify proteins with a similar molecular weight.

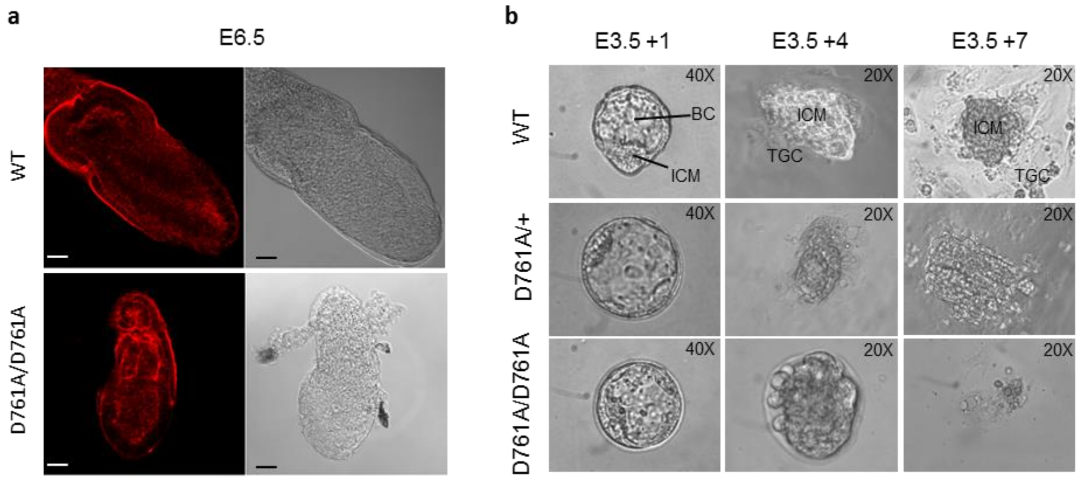

Supplementary Figure 2. Early embryonic lethality and delayed blastocyst outgrowth in Vps34$^{D761A/D761A}$ mice. (**a**) E6.5 embryos were stained for actin using TRITC-phalloidin (red) and imaged using confocal microscopy. Scale bars: 100 µm. (**b**) Blastocysts of the indicated genotypes were explanted at E3.5 and grown in culture for 7 days. Images of explanted blastocysts at 1 (E3.5 + 1), 4 (E3.5 + 4), and 7 (E3.5 + 7) days post-harvest are shown at 20x and 40x magnification, as indicated. Inner cell mass (ICM), blastocoelic cavity (BC) and trophoblast giant cells (TGCs) are indicated on the control.

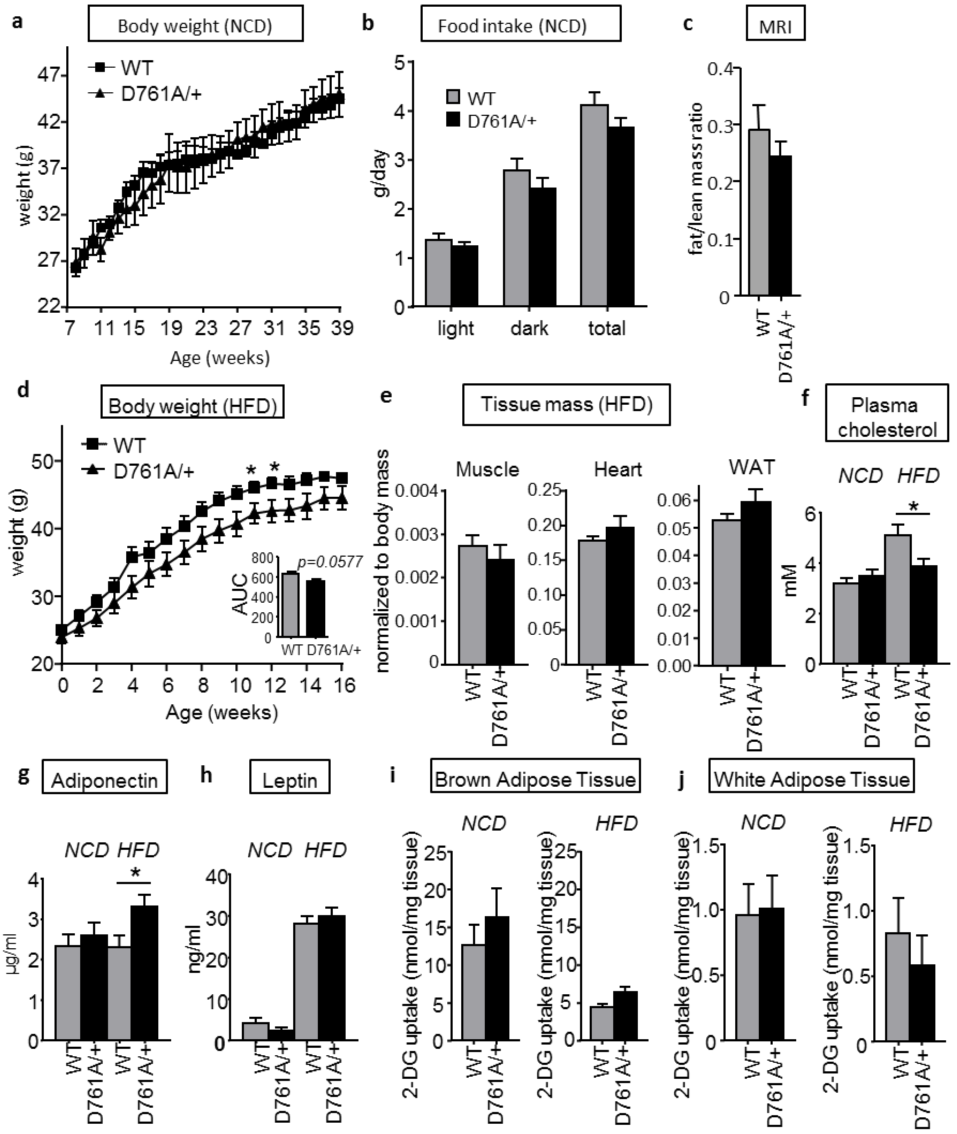

Supplementary Figure 3. Metabolic parameters of Vps34$^{D761A/+}$ mice. (**a**) Body weight of mice fed with normal chow during adult development from 7 to 39 weeks of age. 10 mice/genotype. Data represent mean ± SEM. (non-parametric Mann–Whitney t-test). (**b**) Food intake monitored over two consecutive 24-h cycles for 14-week-old mice kept in 12-h light, 12-h dark conditions and fed with normal chow. 10 mice/genotype. Data represent mean ± SEM. (non-parametric Mann–Whitney t-test). (**c**) Fat and lean mass of 15-week-old mice fed with normal chow. 10 mice/genotype. Data represent mean ± SEM. (non-parametric Mann–Whitney t-test). (**d**) Body weight of mice subjected to 16 weeks of HFD. 10 mice/genotype. Data represent mean ± SEM. (non-parametric Mann–Whitney t-test). (**e**) Comparison of tissue weight of mice fed a HFD normalized to body mass. Data represent mean ± SEM (non-parametric Mann-Whitney t-test). Quantification of 5-9 tissues/genotype. (**f-h**) Plasma cholesterol, adiponectin and leptin levels in mice fed NCD or HFD for 16 weeks. Data represent mean ± SEM. (non-parametric Mann–Whitney t-test) 8 mice/genotype. (**i,j**) Impact of Vps34 inactivation on brown adipose tissue (**i**) and white adipose tissue (**j**) in hyperinsulinemic-euglycemic clamp experiments**.** 5 and 7 mice/genotype for the NCD and HFD studies, respectively. Data represent mean ± SEM. (non-parametric Mann–Whitney t-test). $*p < 0.05$, $**p \leq 0.01$, $***p \leq 0.001$.

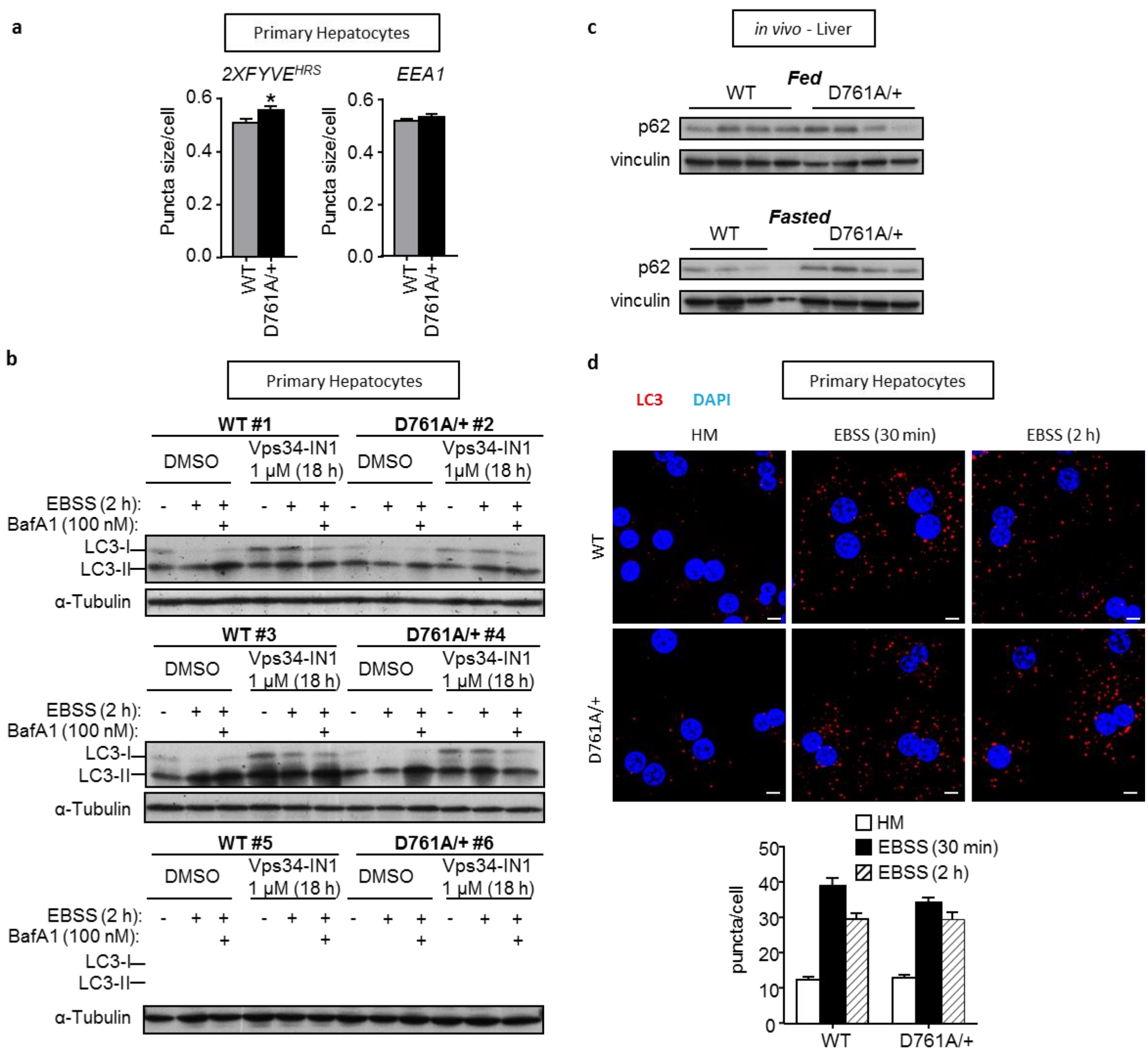

Supplementary Figure 4. Impact of Vps34 inactivation on autophagy in primary hepatocytes. (**a**) Analysis of endogenous PI3P pools in primary hepatocytes. Quantification using Metamorph software of FYVE and EEA1 puncta size represented in confocal images shown in Supplementary Figure 6a. Cell cultures derived from 3-5 independent mice/genotype were used. Data represent mean ± SEM. (non-parametric Mann–Whitney t-test). (**b**) LC3 lipidation assay in primary hepatocytes. Cells were freshly isolated from liver and were grown overnight in HM. Cells were grown in autophagy-induction media (EBSS) in absence or presence of 100 nM Bafilomycin A1 (BafA1) for the indicated times. Cell lysates were immunoblotted with the indicated antibodies. Representative data from 3 independent experiments are shown. (**c**) Immunoblot of p62 levels in liver tissues isolated from mice that were randomly fed or starved for 20 h. Fresh livers were snap-frozen, lysed and immunoblotted with the indicated antibodies. n=4 mice/genotype. (**d**) Upper panel, analysis of LC3 puncta formation (red) upon starvation (EBSS) in primary hepatocytes. Representative data from 3-5 independent experiments. DAPI-stained nuclei are shown in blue. Hepatocytes cultures derived from 3-5 independent mice/genotype were used. HM: Hepatocyte medium. Scale bar, 20 µm. Lower panel, quantification using Metamorph software of LC3 puncta formation in primary hepatocytes. Hepatocytes cultures derived from 3-5 independent mice/genotype were used. HM: Hepatocyte medium. Representative data from 3 independent experiments are shown. Data represent mean ± SEM. (non-parametric Mann–Whitney t-test). $^*p < 0.05$, $^{**}p \leq 0.01$, $^{***}p \leq 0.001$.

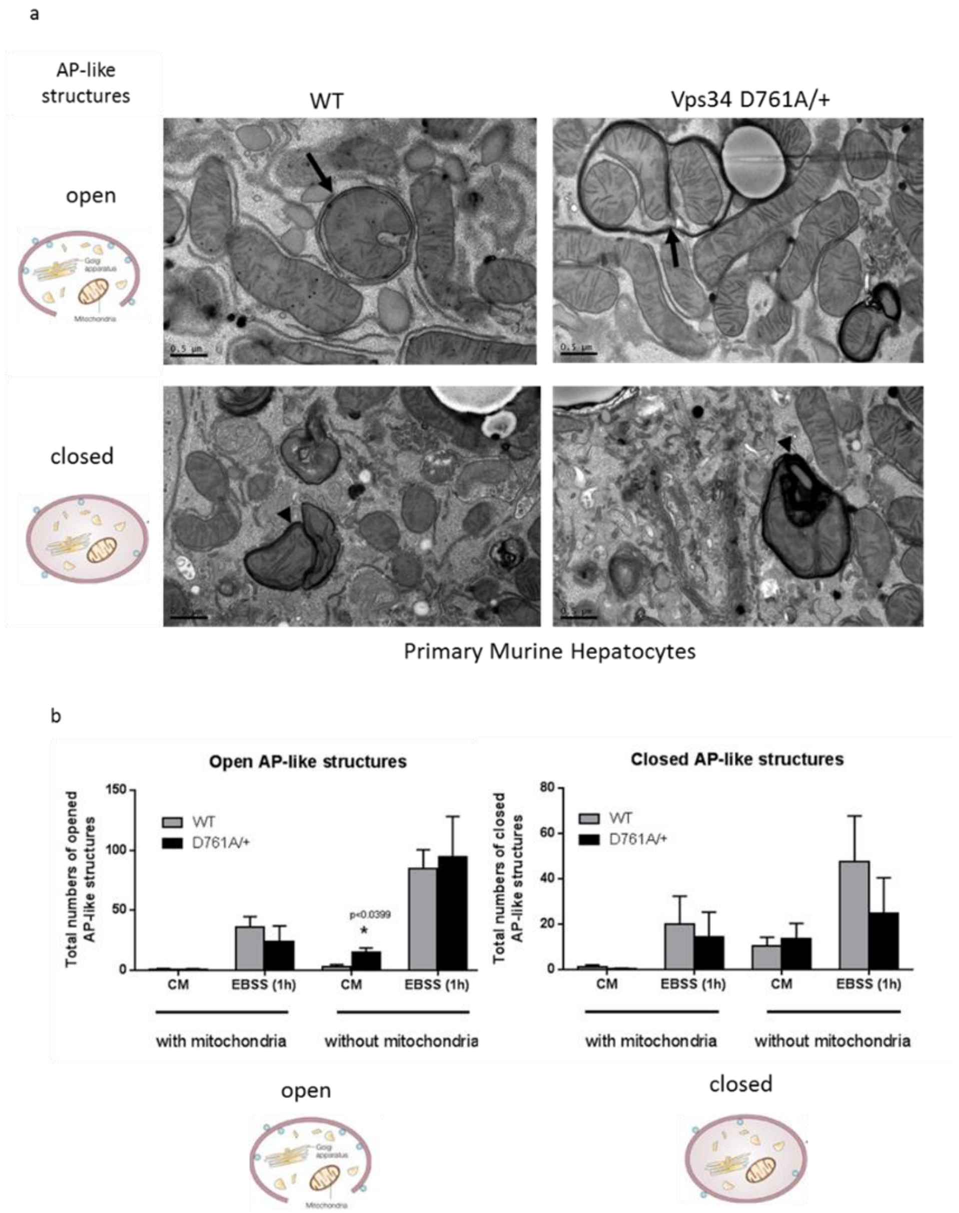

Supplementary Figure 5. Electron microscopy (EM) analysis of WT and Vps34$^{D761A/+}$ primary hepatocytes. (a) Primary murine hepatocytes were cultured in complete medium (CM), fixed, and subjected to conventional EM analysis. Representative images are shown. Arrows depict the open AP-like structures and arrowheads show the closed AP-like structures. Scale bar, 0.5 µm (b) Quantification of the EM images of WT and Vps34$^{D761A/+}$ primary hepatocytes cultured in complete medium (CM) or in starvation medium (EBSS) for 1 h. Data represent mean ± SD (student t-test).

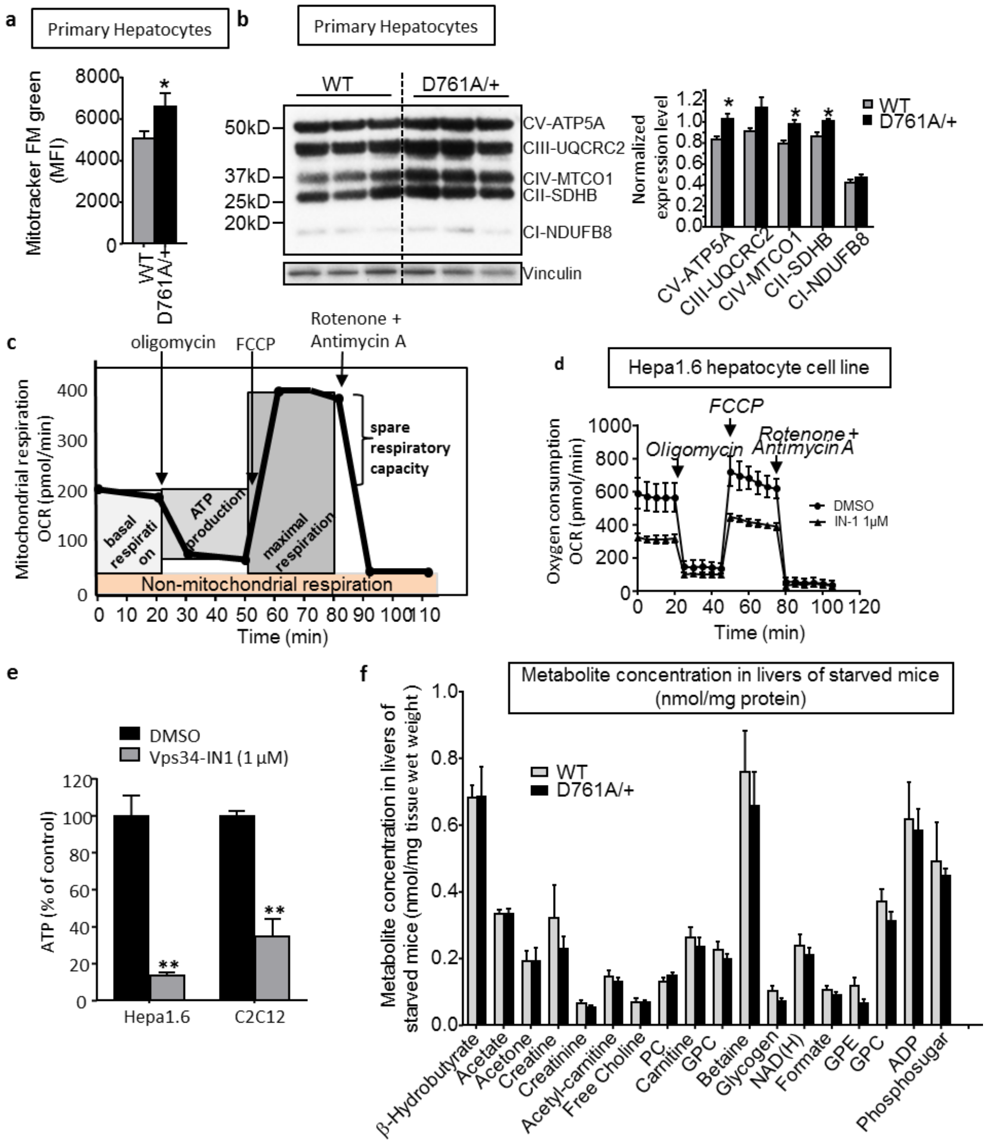

Supplementary Figure 6. Impact of Vps34 inactivation on mitochondrial content and function. (**a**) FACS analysis using the mitotracker FM-dye of viable primary hepatocytes cultured in absence of insulin. MFI: Mean Fluorescent Intensity. Cell cultures derived from 3-5 independent mice/genotype were used. Data represent mean ± SD (student t-test). (**b**) Left panel, representative Western blots of total OXPHOS proteins [from electron transport chain (ETC) complexes I–V] in primary hepatocytes and quantification of the western blots (right panel). Each lane represents a different culture. Data represent mean ± SD (student t-test). (**c**) Measurement of mitochondrial respiration using Seahorse technology. The XF-24 Extracellular Flux analyzer was used to perform the mitochondrial stress test, which measures several parameters of mitochondrial function in cells in real time. A representative experiment is shown in which the basal oxygen consumption rate (OCR) is allowed to stabilize before the sequential addition of oligomycin, FCCP, rotenone and antimycin A. Oligomycin, which inhibits ATP synthesis by blocking complex V, is used to distinguish the percentage of oxygen consumption given over to ATP synthesis and to overcome the natural proton leak across the inner mitochondrial membrane. FCCP uncouples ATP synthesis from the electron transport chain by transporting H^+ ions across the inner mitochondrial membrane. Mitochondrial membrane permeabilization leads to the consumption of both oxygen and energy, without generating ATP, and is used to calculate the spare respiratory capacity (SRC). Rotenone and antimycin A block complex I and III of the electron transport chain, respectively, and shut down mitochondrial respiration, allowing the calculation of both the mitochondrial and non-mitochondrial fractions contributing to respiration. The time course is annotated to show the relative contribution of non-respiratory chain oxygen consumption (basal respiration), ATP-linked

oxygen consumption (ATP production) and the maximal oxygen consumption (maximal respiration) after the addition of FCCP, and the reserve capacity of the cells (spare respiratory capacity). (**d**) Seahorse XF24 measurement of oxygen consumption (OCR) by the Hepa1.6 hepatoma cell line in the absence or in presence of 1 µM Vps34-IN1. Data from 3 independent cultures are shown. (**e**) Decreased ATP levels in Hepa1.6 hepatoma and C2C12 myoblast cells upon Vps34 inactivation. Cells were cultured in presence or absence of 1 µM Vps34-IN1 overnight in starvation media. ATP levels are presented as % of control for each cell line. Data represent mean ± SEM of 3 independent experiments. (unpaired t-test) (**f**) Metabolite concentration in nmol/mg tissue wet weight in fasted liver. Liver from overnight fasted mice were collected, snap-frozen and processed for NMR analysis. 4-5 mice/genotype. Data represent mean ± SD (student t-test). *$p \leq 0.05$, **$p \leq 0.01$, ***$p \leq 0.001$.

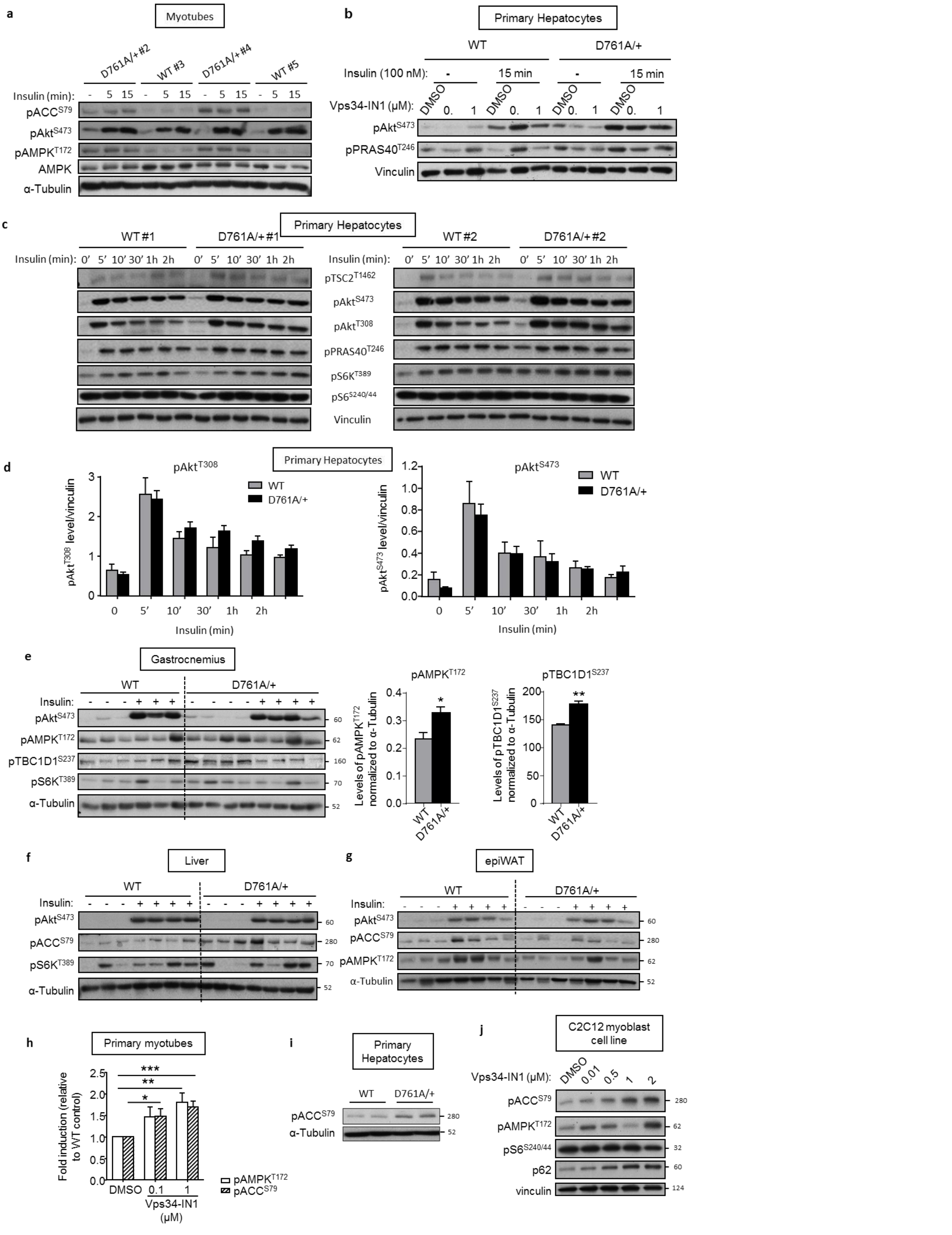

Supplementary Figure 7. Impact of Vps34 inactivation on insulin-mediated Akt signaling. (**a**) Cultured myotubes were starved for 5 h and stimulated for the indicated time points with 100 nM insulin. Cell lysates were immunoblotted with the indicated antibodies. Data representative of 3 independent experiments are shown. (**b**) Primary hepatocytes were cultured overnight in absence of insulin and in presence of DMSO or 1 µM of Vps34-IN1. The next day cells were stimulated with 100 nM insulin for the indicated times. Cell lysates were immunoblotted with the indicated

antibodies. Data representative of 5 independent experiments are shown. (**c**) Primary hepatocytes were cultured overnight in absence of insulin and were stimulated with 100 nM insulin for the indicated times. Cell lysates were immunoblotted with the indicated antibodies. Data representative of 5 independent experiments are shown. (**d**) Densitometric analysis of phosphoprotein levels normalized to the expression level of vinculin from immunoblot experiments on primary hepatocytes. Data represent mean ± SEM (student t-test) of 5 independent experiments. (**e-g**) Tissue homogenates from gastrocnemius muscle (**e**), liver (**f**) and epididymal white adipose tissue (epiWAT) (**g**), isolated from overnight-starved mice 30 min after intraperitoneal injection of 0.75 U/kg insulin or PBS, were immunoblotted using the indicated antibodies. Each lane represents an individual mouse. Quantification of the muscle $pAMPK^{T172}$ and $pTBC1D1^{S237}$, normalized to α-tubulin levels, are shown. (**h**) Quantification of immunoblot performed from lysates of differentiated myotubes treated with DMSO or indicated doses of Vps34-IN1. Data represent mean ± SEM of 5 independent experiments. (non-parametric Mann–Whitney t-test) *p < 0.05, **p ≤ 0.01, ***p ≤ 0.001. (**i**) Primary hepatocytes were cultured overnight in insulin-free HM media and lysates were immunoblotted with the indicated antibodies. Representative data from 6 independent experiments are shown. (**j**) C2C12 myoblast cells were differentiated for 7 days and cultured overnight with DMSO or 1 µM of Vps34-IN1 at the indicated doses. The next day, cells were serum-starved for 5 h. Cell lysates were immunoblotted with the indicated antibodies. Data are representative from 3 independent experiments.

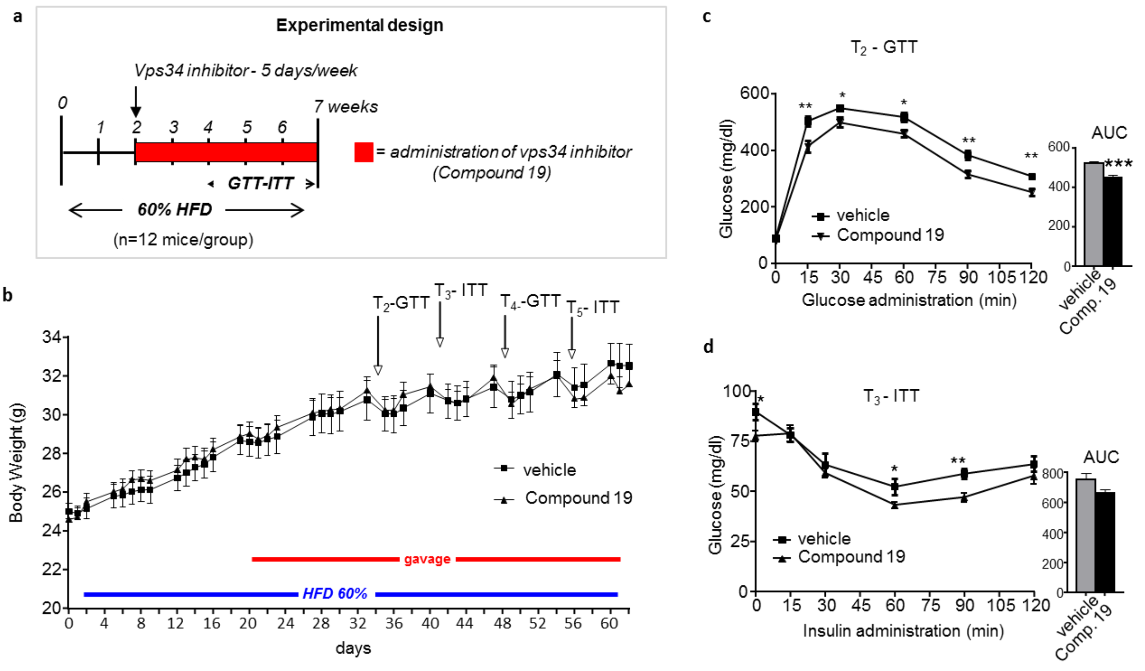

Supplementary Figure 8. Impact of pharmacological inhibition of Vps34 on insulin resistance in HFD-fed mice. (**a**) Experimental design. Mice were subjected to 60% HFD for 7 weeks. Two weeks into the HFD, mice were dosed either with vehicle or Compound 19 (*PO*, Q.D. at 20 mg/kg of mouse weight) for 5 consecutive days a week (Monday to Friday). Two weeks later, mice were starved overnight, followed by a first (‘T_2-GTT’) and a second GTT after 4 weeks (‘T_4-GTT’), and an ITT after 3 weeks (‘T_3-ITT’) and 5 weeks (‘T_5-ITT’), with a week of recuperation between the assays, while still on drug. 10-12 mice were used per group. (**b**) Body weight of mice subjected to 7 weeks of HFD. 10 mice/genotype. Data represent mean ± SEM (non-parametric Mann-Whitney t-test). (**c**) GTT after intraperitoneal injection of 2 g/kg of glucose after overnight starvation in HFD-fed mice treated with Compound 19 for 2 weeks. Data represent mean ± SEM (non-parametric Mann-Whitney t-test) (**d**) ITT after intraperitoneal injection of 0.75 U/kg of insulin after overnight starvation in HFD-fed mice treated with Compound 19 for 3 weeks. The area under the curve (AUC) is shown adjacent to the graph. 10-12 mice per group. Data represent mean ± SEM (non-parametric Mann-Whitney t-test). *$p < 0.05$, **$p \leq 0.01$, ***$p \leq 0.001$.

Supplementary Figure 9. Uncropped immunoblots and larger blot areas of the main figures.

Supplementary Figure 9: related to Fig. 1a

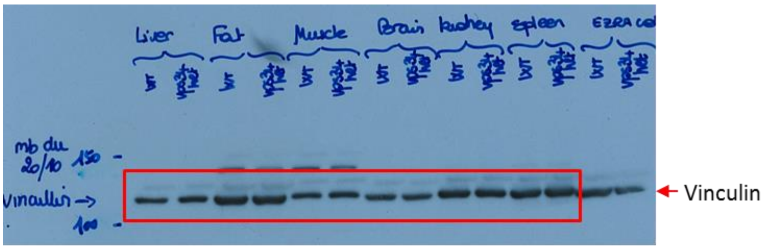

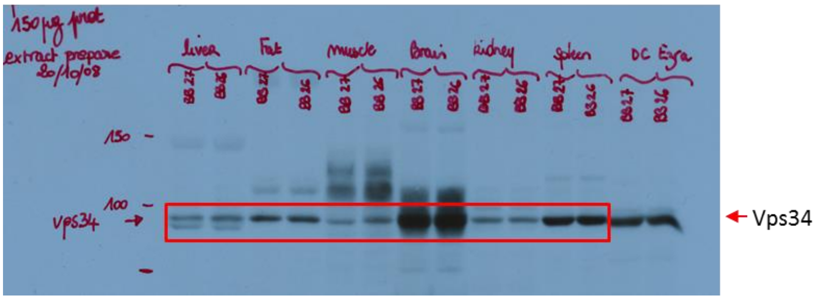

Supplementary Figure 9: related to Fig. 1c

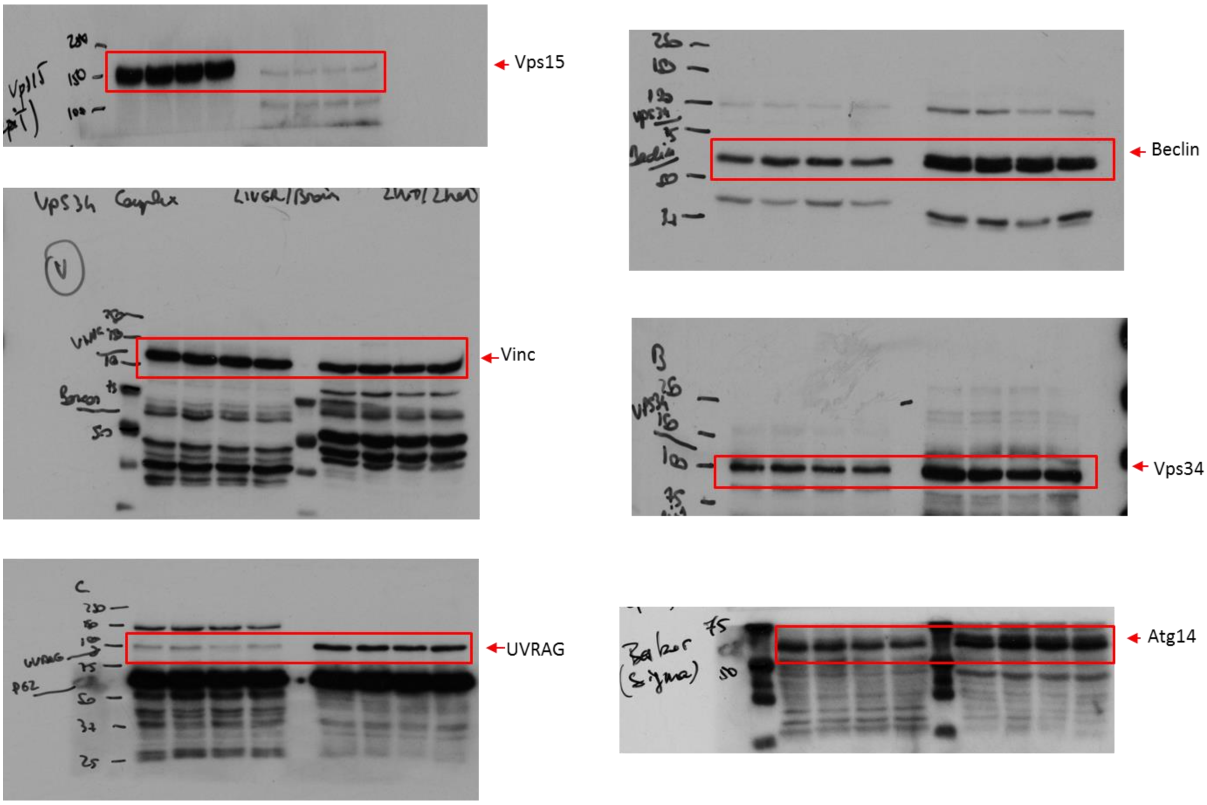

Supplementary Figure 9: related to Fig. 9a

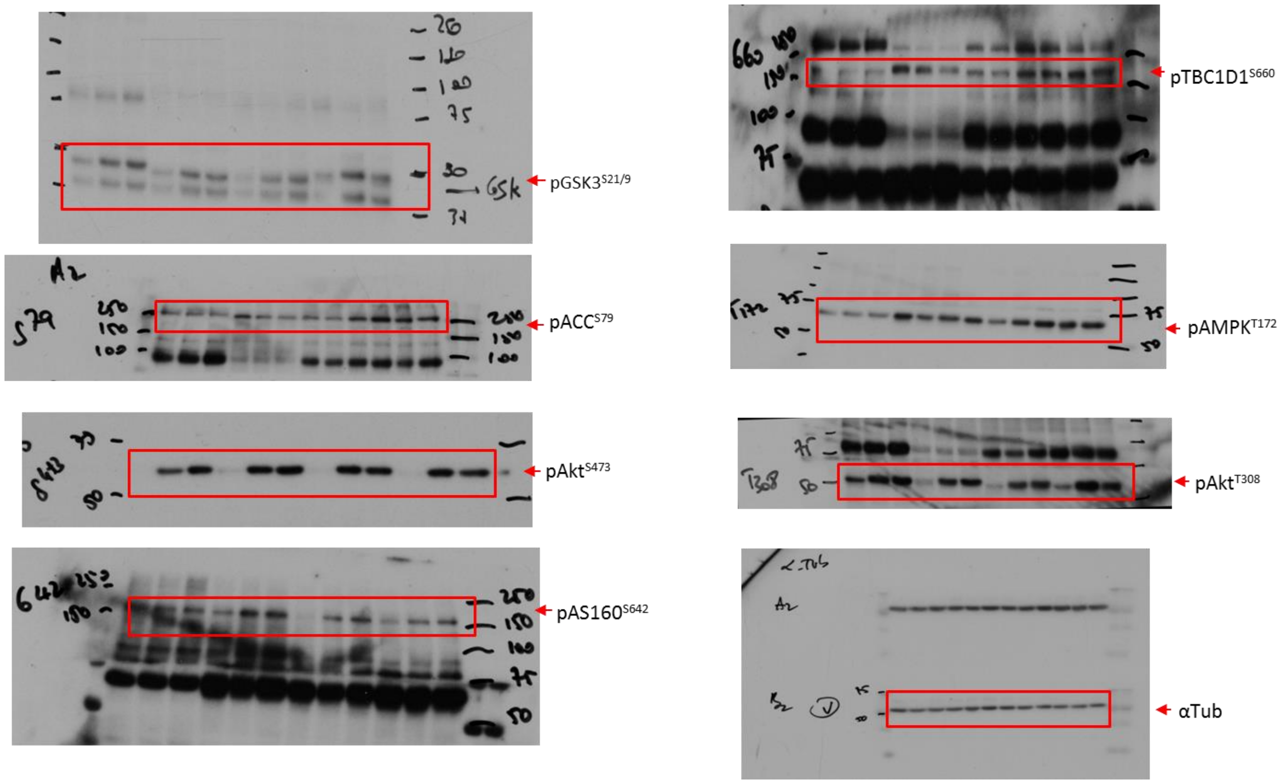

Supplementary Figure 9: related to Fig. 9b

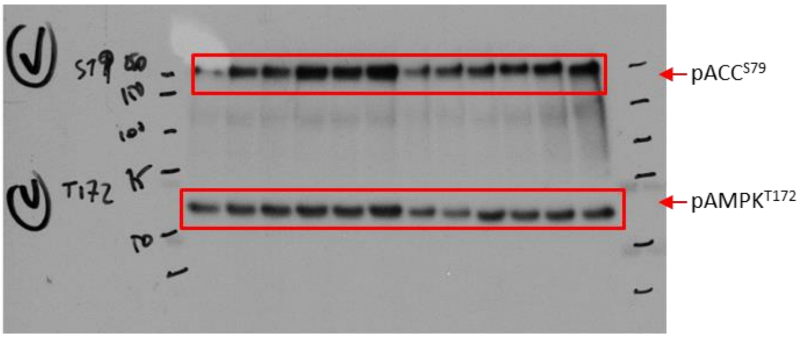

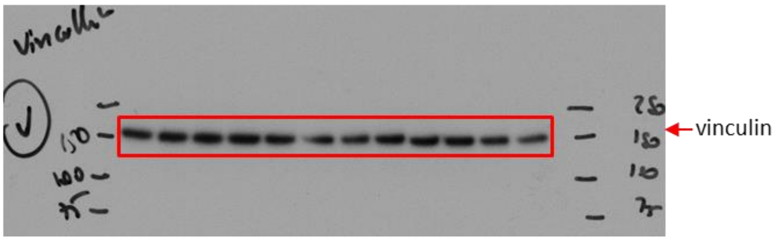

Supplementary Figure 9: related to Fig. 10c

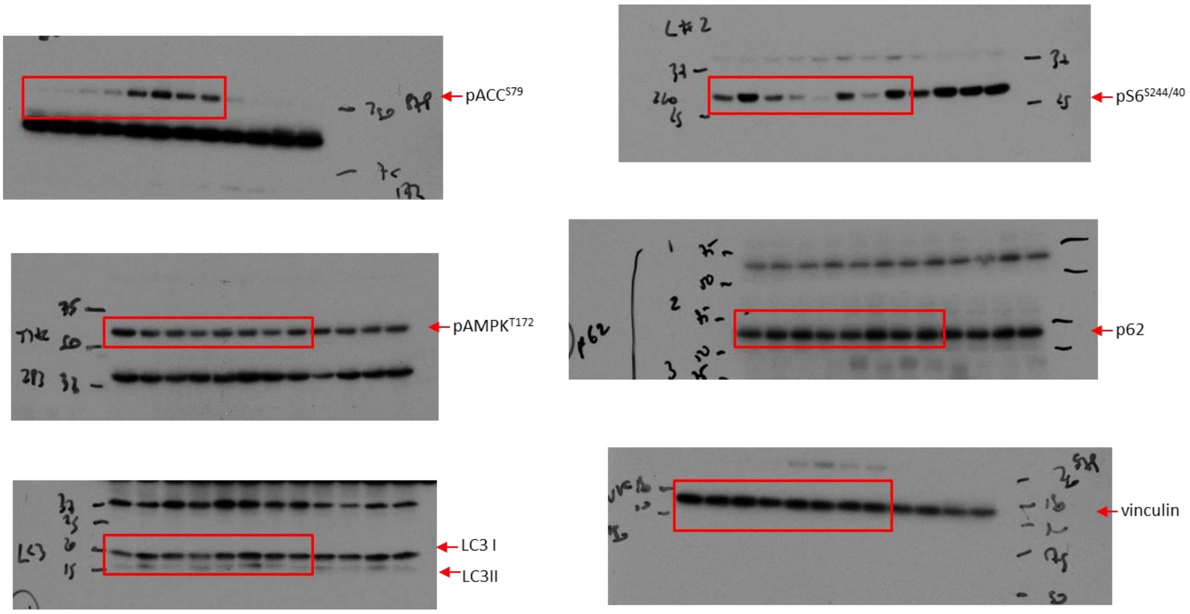

Supplementary Tables

Supplementary Table 1. Number of viable Vps34$^{D761A/D761A}$ embryos obtained at different embryonic stages from intercrosses of Vps34$^{D761A/+}$ mice. (E = embryonic day).

Genotype	E3.5	E5.5	E6.5	E8.5	E9.5	E10.5	E13.5	Born
WT	5	6	4	1	2	8	10	20
Vps34$^{D761A/+}$	8	8	2	5	7	18	20	38
Vps34$^{D761A/D761A}$	4	4	1	0	0	0	0	0

Supplementary Table 2. List of organs and tissues of 17-week-old WT and Vps34$^{D761A/+}$ mice (male) subjected to histological examination (H&E staining).

adrenal	skeletal muscle *(gastrocnemius)*
aorta	optical nerve
brain	pancreas
brown fat	perigenital fat pad
colon	perirenal fat pad
duodenum	pituitary
epidydimes	prostate
oesophagus	salivary gland
eyes	sciatic nerve
femur	skin
gall bladder	spinal cord
Harderian gland	spleen
heart	sternum
jejunum, ileum	stomach
kidney	testis
liver	thymus
lungs	thyroid
mammary glands	urinary bladder
mesenteric lymph nodes	

Supplementary Table 3. Glucose kinetics and other metabolic parameters validating the hyperinsulinaemic-euglycaemic clamp shown in Fig. 4 and Supplementary Fig. S3h,i.

	Normal Chow Diet								
	Basal Rd			Insulin Rd			Insulin GIR		
μmol/kg.min	Mean	*sem*	*t-test*	Mean	*sem*	*t-test*	Mean	*sem*	*t-test*
**WT**	20.90	2.20	0.44	57.01	14.42	0.58	38.65	7.41	0.92
**Vps34$^{D761A/+}$**	18.85	1.37		46.12	5.37		39.97	6.12	

	High Fat Diet								
	Basal Rd			Insulin Rd			Insulin GIR		
	Mean	*sem*	*t-test*	Mean	*sem*	*t-test*	Mean	*sem*	*t-test*
**WT**	12.21	0.73	0.19	27.19	2.06	0.94	18.44	4.69	0.30
**Vps34$^{D761A/+}$**	15.23	1.54		26.90	2.68		27.10	4.19	

Supplementary Table 4. List of antibody used in this study (concentrations/dilutions, as well as catalogue numbers).

Antibody target	Origin	Dilution	Manufacturer	Catalogue Number
**Vps34**	rabbit	1:1000	Cell signalling Technology (CST)	3811S
**Vps34**	rabbit	1:1000	CST	4263S
**LC3**	rabbit	1:1000	CST	2775
**Vps15**	rabbit	1:1000	Epitomics	5638-1
**UVRAG**	mouse	1:1000	MBL	160-3
**Beclin-1**	rabbit	1:1000	CST	3738
**ATG14**	rabbit	1:1000	ProteinTech	19491
**Vinculin**	mouse	1:5000	Sigma	9131
**A-Tubulin**	mouse	1:5000	Sigma	T6074
**EEA1 (C45B10)**	rabbit	1:1000	CST	3288
**GST**	rabbit	1:1000	CST	2622
**pAkt (S473)**	rabbit	1:1000	CST	9271
**pAkt (T308)**	rabbit	1:1000	CST	9275
**pAMPK (T172)**	rabbit	1:1000	CST	2531
**pACC (S79)**	rabbit	1:1000	CST	3661
**pPRAS40 (T246)**	rabbit	1:1000	CST	2640
**pTSC2 (T1462 5B12)**	rabbit	1:1000	CST	3617
**pS6K (T389)**	rabbit	1:1000	CST	9205
**pS6 (S240/44)**	rabbit	1:1000	CST	2215
**pTBC1D1 (S660)**	rabbit	1:1000	CST	6928
**pAS160 (T642)**	rabbit	1:1000	CST	4288
**pGSK3 (S21/9)**	rabbit	1:1000	CST	8566
**Tom20 FL145**	rabbit	1:1000	Santa Cruz	sc-11415
**p62 (2C11)**	mouse	1:1000	Novus Biological	H0008878-M01
**Atg14/Barkor**	rabbit	1:1000	Sigma	A6358
**LC3 (2G6)**	mouse	1:1000	Nanotools	0260-100/LC3-2G6
**pTBC1D1 (S237)**	rabbit	1:1000	Millipore	07-2268
**total OXPHOS cocktail Antibody**	mouse	1:1000	Abcam	ab110413

Supplementary Methods

Cell lines

Cells were cultured in a humidified incubator at 37°C and 5% CO_2. The murine Hepa1.6 hepatoma and C2C12 myoblast cell lines were obtained from American Type Culture Collection and maintained in complete medium, defined as follows: Dulbecco's modified Eagle's medium (DMEM) supplemented with 10% foetal bovine serum (FBS), 2 mM L-Glutamine, 50 U/ml penicillin/streptomycin. To differentiate C2C12 cells into myotubes, cells were allowed to grow to about 90% confluence and then were switched to DMEM supplemented with 2% horse serum and refed with this medium every day.

Determination of PI3P in tissue and cell extracts

Total PI3P levels were quantified by a mass assay as described in Ref[1]. Briefly, after removing media, cells were immediately scraped on ice with ice-cold 1 M HCl, followed by centrifugation at 2000 rpm at 4°C and snap-freezing of the cell pellet. Samples were stored at -80°C before processing for the PI3P mass assay. For mass assay on tissues, 50 mg of frozen liver or muscle was homogenized in chloroform/methanol/water (1/2/0.8; v/v/v) in FastPrep homogenizer. Total lipid extraction was performed according to the Bligh and Dyer procedure[2]. PtdInsP were then purified by thin layer chromatography. Lipid kinase assays were performed using recombinant PIKfyve (for PtdIns3P) and [γ-^{32}P]ATP (Perkin Elmer). The radioactive biphosphates generated (proportional to the PtdIns3P present in the sample) were quantified using Typhoon Imaging System (GE Healthcare).

Mouse embryonic fibroblast (MEF) isolation and culture

Cells were cultured in a humidified incubator at 37°C and 5% CO_2. MEFs were isolated as described[3] from intercrosses of heterozygous Vps34$^{D761A/+}$ mice. Briefly, the day of the presence of a copulation plug was considered as day E0.5. Each E13.5 embryos were separated from its placenta and surrounding membranes. Cut away brain and dark red organs were minced, dissociated using trypsin and cells allowed to adhere on tissue culture dishes in DMEM, 10%FBS, penicillin and streptomycin. Unless otherwise indicated, early 500 passage (P2 to P3) MEFs were used in experiments.

Blastocyst isolation and embryo isolation at E6.5

Blastocysts were isolated from Vps34$^{D761A/+}$ intercrosses. E3.5 blastocysts from natural matings were harvested into 96-well plates. E3.5 blastocysts outgrowth was performed as follows: embryos were cultured without LIF in DMEM, 15% fetal bovine serum for a total of 2–5 days and examined by phase contrast microscopy prior to genotyping by PCR. For isolation of embryos at E6.5, embryos were dissected by hand in M2 medium (Sigma), fixed in 4% paraformaldehyde in PBS for 30 min, washed 3x with PBS-0.1% Triton-X100 (PBT), permeabilized in PBS-0.5% Triton-X100 for 15 min, followed by staining with TRITC-Phalloidin (Sigma), four 5-min washes in PBT and mounting in Vectashield mounting media containing DAPI (Vector Labs H-1200). Fixed samples were imaged on a Zeiss LSM 710 confocal microscope using a 40X/1.3NA objective.

Real-time PCR analysis of gene expression

RNA was extracted using RNeasy kit (Qiagen) from the liver of 12 h-fasted mice or starved Hepa1.6 cell lines treated or not with Vps34-IN1. Real-time quantitative PCR reactions were carried out using the Taqman® gene expression assay Universal PCR Master Mix (Applied Biosystems, Foster City, CA) and an ABI Prism 7900 Sequence Detection System (Applied Biosystems, Melbourne, Australia). Values for mRNA transcripts were normalized to the levels of β-actin, given that the levels of β-actin mRNA transcripts were not different between control and Vps34 inhibited mice/cells.

Measurement of glucose uptake

Myotubes were grown in 12-well culture plates and glucose uptake was performed in triplicate on day 7 of differentiation. Before the assay, the differentiation medium was removed from the cell monolayers, and monolayers washed three times with starvation medium (DMEM/F12 + GlutaMAX-1 supplemented with 0.2% BSA) followed by incubation in 1 ml starvation medium for 5 h in 5% CO_2 at 37°C. The monolayers were washed twice with uptake buffer (140 mM NaCl, 20

mM HEPES pH 7.4, 2.5 mM $MgSO_4$, 1 mM $CaCl_2$, 5 mM KCl) and then incubated in 1 ml uptake buffer with or without 100 nM insulin or 20 µM cytochalasin B (Sigma) for the indicated times. Measurement of uptake was initiated by the addition of 2-deoxy-D-glucose, 2-[1,2-3H(N)] (Perkin Elmer). The final concentrations were 1 mM 2-deoxyglucose and 0.5 µCi/ml of 2-deoxy-D-glucose, 2-[1,2-3H(N)]. After incubation for 10 min, glucose uptake was terminated by rapidly washing the culture plates five times with ice-cold PBS, followed by lysis in 0.05 N NaOH for 30 min, followed by liquid scintillation counting. Total cellular protein was determined by the Bradford method. Non-specific glucose uptake was determined in parallel incubations for each individual cell line by exposing the cells to 20 µM cytochalasin B.

AICAR tolerance test

$Vps34^{D761A/+}$ and WT mice were fasted for 12 h prior to the AICAR tolerance test. Mice were randomized and the test was performed blinded. Mice received AICAR (0.5 g/kg body weight) by intraperitoneal injection. Blood was collected from the tail vein and blood glucose concentration was measured via a glucometer.

Histology

For tissue sections, hematoxylin and eosin (H&E) staining was performed on 5 µm paraffin sections of tissues fixed for 24 h in 4% phosphate-buffered paraformaldehyde (PFA) at 4°C. For Oil Red O staining, liver tissue was frozen in OCT, sectioned, and stained using Oil Red O (Sigma-Aldrich) according to standard procedures. In brief, sections were fixed with 4% PFA in PBS at room temperature for 15 min. Fixed sections were washed again with PBS and stained with Oil Red O (0.5% w/v isopropanol, diluted 3:2 in PBS) for 1 h at room temperature. Stained sections were rinsed in 60% isopropanol, followed by deionized water and mounted in Vectashield (Vector Laboratories).

Measurement of ATP concentrations in tissues by UPLC-MS/MS

8-Chloro adenosine triphosphate (ChloroATP) and ATP were from Sigma Aldrich (UK). LC-MS grade water, methanol, acetonitrile and formic acid were from Fisher Scientific, UK.

Sample extraction: Tissues were dissected and immediately snap-frozen in liquid nitrogen. Frozen tissue was weighed and grounded into powder using tissue grinder. 50 mg tissue powder was solubilized in 100 µl of PBS. Another 100 µl of 0.8 M perchloric acid was added to the reconstituted extract; vortex mixed and kept on ice for 30 min. After centrifugation (10,000*g*, 4°C for 10 min), 180 µl of the supernatant was transferred to new Eppendorf tube and kept at -80°C until time of analysis. At the time of analysis, 50 µl of 1 M NH_4Ac was added to the perchloric acid extract, and the solution was neutralised by addition of 20 µl of 10% ammonia solution. Finally, 5 µl containing the internal standard 8-ChloroATP and 5 µl deionised water was added. The extract was transferred to LC-MS vials and 10 µl was injected in to the UPLC-MS/MS system.

***Sample analysis*:** 10 mM stock solutions of ATP were prepared and aliquots frozen at -80°C until use. Fresh calibration standards were prepared by diluting the stock solution in PBS to the following concentrations: 0, 0.1, 0.3, 1, 3, 10, 30 and 100 µM. High and low control samples (5 and 50 µM) were also prepared. The calibration standards and controls were added instead of 5 µl deionised water in the previously described sample extraction method.

***Chromatography and mass spectrometry*:** Analytes were resolved using an ultra-performance liquid chromatography system (Accela UPLC, Thermo Scientific, UK) equipped with a Biobasic AX 5 µm, 50×2.1 mm column (Thermo Electron Corporation, Murrieta, CA, USA) and a mobile phase consisting of a mixture of 10 mM NH_4Ac in ACN/H_2O (30:70 v/v), pH 6.0 (A), and 1 mM NH_4Ac in ACN/H_2O (30:70 v/v), pH 10.5 (B). The mobile phase gradient was employed, comprising: buffer A = 95% at 0 - 0.5 min, from 95 to 0% over 1.25 min, held at 0% for 1.75 min, from 0 to 95% over 0.1 min, ending with 95% for 2.9 min, all at a flow rate of 500 µl/min. Eluting compounds of interest were detected using a triple stage quadrupole Vantage mass spectrometry system (Thermo Scientific, UK) equipped with an electrospray ion source. Samples were analyzed in the Multiple Reaction Monitoring (MRM), negative ion modes at a spray voltage of 3000 V. Nitrogen was used as sheath and auxiliary gas at a flow rate of 50 and 20 arbitrary units, respectively. Argon was used as collision gas with pressure of 1.5 mTorr. The optimum transitional daughter ions mass and collision energy of each analyst were as follows: ATP 506.0 $\rightarrow$ 408.2 (collision energy 23 V and ChloroATP 539.9 $\rightarrow$ 442.2 (collision energy 24 V).

Measurement of ATP concentrations in cell lines

Hepa1.6 and C2C12 cells were incubated overnight with 1 µM Vps34-IN1 or DMSO control in starvation media. The next day, intracellular ATP levels were measured using ATP Bioluminescence Assay kit HS II (Roche) according to the manufacturer's instructions. Values were normalized as a % of control cells.

^{1}H- and ^{31}P-magnetic resonance spectroscopy (MRS) of liver extracts

Freeze-clamped liver samples were extracted by dual-phase extraction procedures as described [4,5]. Briefly, water-soluble extracts were freeze-dried and reconstituted in 650 µl deuterated water (D_2O, Sigma Aldrich) and the extracts (500 µl) were then placed in 5 mm NMR tubes. 50 µl of 0.75% sodium 3-trimethylsilyl-2,2,3,3-tetradeuteropropionate (TSP) in D_2O (Sigma Aldrich) was added to the samples for chemical shift calibration and quantification. ^{1}H- and ^{31}P-MRS were performed on a Bruker 500 MHz spectrometer (Bruker Biospin, Ettlingen, Germany). ^{1}H-MR spectra of the extracted samples were acquired at 25°C using a pulse acquire MR sequence with water suppression (1D NOESY preset sequence) with 7500 Hz spectral width, 32768 time domain points, 2.7 sec repetition time and 512 scans. For ^{31}P MRS, which was carried out after the ^{1}H MRS study, EDTA (50 µl, 60 mM) was added to each sample to chelate metals ions, and methylene diphosphonic acid (50 µl, 5 mM) was added to each sample for chemical shift calibration and quantification. ^{31}P-MR spectra were acquired using an inverse-gated ^{1}H decoupled 31P-MR sequence with 12100 Hz spectral width, 32768 time domain points, 5 sec repetition time and 4000 scans. Spectral assignments were based on literature [4,6].

Isolation of mitochondria from liver

Mitochondria were isolated by subcellular fractionation, as described[7]. Briefly, mice were sacrificed by cervical dislocation, the liver removed, and placed into ice-cold isolation buffer (250 mM mannitol, 5 mM HEPES pH7.4, 0.5 mM EGTA). The liver was rinsed on ice with cold PBS to remove excess blood, followed by removal of connective tissue and fat. The liver was then placed in isolation buffer containing PMSF (1 mM), diced into small pieces of approximately 2 mm width, followed by homogenization using a Dounce homogenizer with an electronic pestle, until no solid matter remained, and centrifuged at 4°C for 10 min at 800g. This gave rise to a nuclear pellet, which was discarded, and a post-nuclear supernatant, which was retained and centrifuged at 4°C for 10 min at 10300g. The resulting (post-mitochondrial) supernatant was then discarded, and the mitochondrial pellet was resuspended in isolation buffer and PMSF (1 mM) and kept on ice until use. Protein levels were quantified using the ThermoScientific BCA protein quantification assay (ThermoScientific) according to manufacturer's instructions.

Measurement of mitochondrial respiration with High Resolution Oxygraph

Isolated mitochondria were resuspended in respiration buffer (110 mM sucrose, 0.5 mM EGTA, 3.0 mM $MgCl_2$, 80 mM KCl, 60 mM K-lactobionate, 10 mM KH_2PO_4, 20 mM taurine, 20 mM HEPES pH 7.1, 1.0 g/l BSA) at a concentration of 0.8 mg/ml and respiration rates were measured at 37°C with the High Resolution Oxygraph OROBOROS (Oxygraph-2k, Graz, Austria). State 3 oxidative capacities were determined in the presence of saturating concentrations of oxygen, ADP (0.25 mM) and specific mitochondrial substrates. For complex I-dependent respiration, substrates were glutamate (10 mM) malate (5 mM) and pyruvate (1 mM). For measurement of complex II-dependent respiration, rotenone (0.5 µM) and succinate (10 mM) were used. Oxygen consumption is expressed as pmol O_2/min/µg mitochondrial protein.

Measurements of oxygen consumption and extracellular acidification

Oxygen consumption rate (OCR) and extracellular acidification rate (ECAR) were measured using a Seahorse XF-24 Flux Analyzer (Seahorse Biosciences, North Billerica, MA). Primary hepatocytes were seeded at 4×10^{4} cells/well in Seahorse XF 24-well tissue culture plates precoated with collagen. Primary myoblasts were seeded at 1×10^{4} cells/well in Seahorse XF 24-well tissue culture precoated with Matrigel. Hepatocytes were cultured for 24 h in HM and primary myotubes for 7 days in differentiation medium. Prior to the assay, the medium was replaced by unbuffered DMEM containing 0.5 mM pyruvate (Gibco, pH 7.4 at 37°C) and the cultures equilibrated for 60 min at 37°C. The following agents were sequentially injected during the assay (final concentration

between brackets): oligomycin (1 µM for hepatocytes and 2 µM for myotubes), FCCP (0.5 µM), antimycin A (1 µM) or rotenone (1 µM). OCR and ECAR were measured for 100 min at 2 min intervals. The OCR and ECAR for hepatocytes or myotubes were then normalized to total protein concentration after the assay was completed.

Hyperinsulinemic clamp

Clamp experiments were performed as described[8-14] after an overnight fast. Briefly, animals were anesthetized by intraperitoneal injection of a combination of 6.25 mg/kg acetylpromazine (Sanofi Santé Nutrition Animale, LibourneCedex, France), 6.25 mg/kg midazolam (Roche, Mijdrecht, The Netherlands), and 0.31 mg/kg fentanyl (Janssen-Cilag, Tilburg, The Netherlands). An infusion needle was placed into the tail vein. After 50 min infusion of D-[3-3H]glucose at a rate of 0.006 MBq/min (Glucose, D-[3-3H], Specific Activity: 10-20 Ci(370-740 GBq)/mmolPerkin-Elmer, UK) to achieve steady-state levels, basal parameters were determined with 10 min intervals (B1 and B2). Thereafter, a bolus of insulin (3.3 mU, Actrapid; Novo Nordisk, Chartres, France) was administered and the hyperinsulinemic clamp was started. Insulin was infused at a constant rate of 0.09 mU/min, and D-[3-3H]glucose was infused at a rate of 0.006 MBq/min. A variable infusion of 12.5% D-glucose (in PBS) was started to maintain blood glucose at basal levels (euglycemic) Blood glucose was measured with a AlphaTRAK hand glucose-meter (Abbott Animal Health, Netherlands) every 5-10 min and accordingly adjust the glucose infusion pump. 50 min after initiating the insulin infusion an i.v. bolus of ^{14}C-labelled 2-deoxy-D-Glucose (deoxy-D-glucose, 2-[14C(U)], Specific Activity: 250-350 mCi (9.25-13.0GBq)/mmol, Perkin-Elmer, UK) was given for assessment of tissue specific glucose uptake. After 90 min reaching steady state, blood samples were taken at 10 min intervals during 30 min to determine steady-state levels of [^{3}H]glucose. After the last blood sample, mice were killed by cervical dislocation and the organs were dissected and immediately frozen. An average clamp experiment took approximately 3 h, and anaesthesia was maintained throughout the procedure. Haematocrit was measured at baseline and after the clamp. No relevant alterations where observed between the different genotypes (data not shown).

***Analysis of clamp samples*:** Plasma insulin concentrations were measured by ELISA (Crystal Chem Downers Grove, USA). To measure plasma [^{3}H]glucose, trichloroacetic acid (final concentration 2%) was added to 10 µl plasma to precipitate proteins using centrifugation. The supernatant was dried to remove water and re-suspended in water. The samples were counted using scintillation counting (HidexScintilation counter, LabLogic, Sheffield, UK).

Calculations: The glucose turnover rate (µmol/min) was calculated during the basal period and under steady-state clamp conditions as the rate of tracer infusion (dpm/min) divided by the plasma specific activity of [^{3}H]glucose (dpm/µmol). The hyperinsulinemic hepatic glucose production was calculated as the difference between the tracer-derived rate of glucose appearance and the glucose infusion rate.

***Tissue analysis*:** Tissue samples were homogenized (~5-10% wet wt/vol, depending on tissue) in 0.5% percholic acid and centrifugated. Supernatants were neutralized and 2DG-P was precipitated using BaOH/$ZnSO_4$. Total and precipitated counts of supernatants were subtracted to give tissue specific 2-deoxy-D-Glucose uptake. Plasma ^{14}C-2-deoxy-D-Glucose specific activity was used to calculate tissue specific uptake. Protein content in homogenates was performed using DC protein assay (BioRad, UK).

Quantification of autophagosome-like structures in cultured hepatocytes

Primary hepatocytes were fixed in 2.5% glutaraldehyde/4% paraformaldehyde in 0.1 M PB for 60 min. After fixation, the coverslips were washed several times in 0.1 M PB and post-fixed in 1.5% potassium ferricyanide/1% osmium tetroxide for 1 h, prior to further washing in PB and incubation in 1% tannic acid in 0.05 M PB for 45 min to enhance membrane contrast. After a brief incubation in 1% sodium sulphate in 0.05 M PB, the coverslips were washed twice in distilled water, and dehydrated through an ascending series of Ethanol (30%, 50%, 2x70%, 2x90%, 2x100%), and propylene oxide before infiltration with Epoxy resin and polymerisation overnight at 60°C. The coverslips were removed from the resin blocks by plunging briefly into liquid nitrogen. The polymerised blocks were trimmed by hand using a single edged razor blade to form a trapezoid block face for serial ultrathin sectioning. Using a diamond knife, serial ultrathin sections of approximately 70 nm thickness were collected on 1% formvar coated single slot grids. The sections were counterstained with lead citrate to further enhance contrast prior to viewing in the

electron microscope (FEI Tecnai G2 Spirit BioTWIN with Gatan Orius CCD camera). For quantification of ‘open’ and ‘closed’ autophagosome-like structures, cells were chosen at random by placing a 20x20 grid over a low magnification image of a single section, and using a random number generator to select individual grid squares. The entirety of a cell intersecting the chosen square was imaged. The imaging conditions and depth of section (with respect to distance from the coverslip surface) were kept consistent between samples. All autophagosome-like structures in each of 5 cells from WT and Vps34 $^{D761A/+}$ cultures (n=3 for each) were examined. The autophagosome-like structures were classified as ‘open’ when the contents of the structure were similar in appearance to the surrounding cellular cytoplasm; ‘closed’ structures displayed a distinct difference in the density and/or granularity of their contents when compared to the surrounding cytoplasm. The grid overlay for cell selection and viewing was done using ImageJ software. All data are shown as mean ± SEM. Data sets were compared for statistical significance using two-tailed Student’s test as: *$p < 0.05$, **$p \leq 0.01$, ***$p \leq 0.001$.

Statistical analysis

All data are shown as mean ± SEM. Data sets were compared for statistical significance using Mann-Whitney test or two-tailed Student’s test as indicated in the figure legend. All statistical analyses were generated using the GraphPad Prism 6 software and statistical significance indicated as: *$p < 0.05$, **$p \leq 0.01$, ***$p \leq 0.001$.

Supplementary References

1. Chicanne, G.*, et al.* A novel mass assay to quantify the bioactive lipid PtdIns3P in various biological samples. *Biochem J* **447**, 17-23 (2012).
2. Bligh, E.G. & Dyer, W.J. A rapid method of total lipid extraction and purification. *Canadian journal of biochemistry and physiology* **37**, 911-917 (1959).
3. Foukas, L.C.*, et al.* Critical role for the p110alpha phosphoinositide-3-OH kinase in growth and metabolic regulation. *Nature* **441**, 366-370 (2006).
4. Chung, Y.L.*, et al.* Magnetic resonance spectroscopic pharmacodynamic markers of the heat shock protein 90 inhibitor 17-allylamino,17-demethoxygeldanamycin (17AAG) in human colon cancer models. *J Natl Cancer Inst* **95**, 1624-1633 (2003).
5. Tyagi, R.K., Azrad, A., Degani, H. & Salomon, Y. Simultaneous extraction of cellular lipids and water-soluble metabolites: evaluation by NMR spectroscopy. *Magn Reson Med* **35**, 194-200 (1996).
6. Sitter, B., Sonnewald, U., Spraul, M., Fjosne, H.E. & Gribbestad, I.S. High-resolution magic angle spinning MRS of breast cancer tissue. *NMR in biomedicine* **15**, 327-337 (2002).
7. Astin, R.*, et al.* No evidence for a local renin-angiotensin system in liver mitochondria. *Scientific reports* **3**, 2467 (2013).
8. Voshol, P.J.*, et al.* In muscle-specific lipoprotein lipase-overexpressing mice, muscle triglyceride content is increased without inhibition of insulin-stimulated whole-body and muscle-specific glucose uptake. *Diabetes* **50**, 2585-2590 (2001).
9. Kreier, F.*, et al.* Selective parasympathetic innervation of subcutaneous and intra-abdominal fat--functional implications. *The Journal of clinical investigation* **110**, 1243-1250 (2002).
10. Netea, M.G.*, et al.* Deficiency of interleukin-18 in mice leads to hyperphagia, obesity and insulin resistance. *Nat Med* **12**, 650-656 (2006).
11. Gray, S.L.*, et al.* Leptin deficiency unmasks the deleterious effects of impaired peroxisome proliferator-activated receptor gamma function (P465L PPARgamma) in mice. *Diabetes* **55**, 2669-2677 (2006).
12. Bouskila, M.*, et al.* Allosteric regulation of glycogen synthase controls glycogen synthesis in muscle. *Cell Metab* **12**, 456-466 (2010).
13. Stienstra, R.*, et al.* The inflammasome-mediated caspase-1 activation controls adipocyte differentiation and insulin sensitivity. *Cell Metab* **12**, 593-605 (2010).
14. Chow, S.Z.*, et al.* Glycoprotein 130 receptor signaling mediates alpha-cell dysfunction in a rodent model of type 2 diabetes. *Diabetes* **63**, 2984-2995 (2014).
